# Prevalence of undernutrition and associated factors in young children in Malaysia: A nationwide survey

**DOI:** 10.3389/fped.2022.913850

**Published:** 2022-08-18

**Authors:** Way Seah Lee, Muhammad Yazid Jalaludin, Kim Mun Khoh, Juan Loong Kok, Thiyagar Nadarajaw, Anna Padmavathy Soosai, Firdaus Mukhtar, Yong Junina Fadzil, Azriyanti Anuar Zaini, Siti Hawa Mohd-Taib, Rozanna M. Rosly, An Jo Khoo, Hon Kit Cheang

**Affiliations:** ^1^Department of Paediatrics, Faculty of Medicine, University Malaya, Kuala Lumpur, Malaysia; ^2^Faculty of Medicine and Health Sciences, University Tunku Abdul Rahman, Kajang, Malaysia; ^3^Paediatric Unit, Gleneagles Medical Centre, Kota Kinabalu, Sabah, Malaysia; ^4^Paediatric Unit, Sarawak General Hospital, Kuching, Sarawak, Malaysia; ^5^Paediatric Unit, Hospital Sultanah Bahiyah, Alor Setar, Kedah, Malaysia; ^6^Prince Court Medical Centre, Kuala Lumpur, Malaysia; ^7^Faculty of Medicine and Health Sciences, Universiti Putra Malaysia, Serdang, Selangor, Malaysia; ^8^Klinik Pakar Kanak-Kanak Junina, Petaling Jaya, Selangor, Malaysia; ^9^Department of Dietetics, University Malaya Medical Centre, Kuala Lumpur, Malaysia; ^10^Dietetic Services Department, UM Specialist Centre, Kuala Lumpur, Malaysia; ^11^Department of Dietetics and Food Service, Lam Wah Ee Hospital, Georgetown, Penang, Malaysia; ^12^Paediatric Unit, Lam Wah Ee Hospital, Georgetown, Penang, Malaysia

**Keywords:** prevalence, risk factors, young children, at risk of undernutrition, undernutrition, stunting, wasting

## Abstract

**Introduction:**

Undernutrition in young children is a significant public health problem globally. We determined the prevalence of and factors predisposing to stunting and underweight in children aged 1 to 5 years in Malaysia.

**Materials and methods:**

Data were extracted from a cross-sectional nationwide campaign involving healthy children aged 1–5 years conducted over a 4-month period in 2019. We obtained information on demography, parental height and risk factors of undernutrition and anthropometric measurements (height and weight) of children enrolled. Age and sex-specific z-score for length/height-for-age (HAZ), weight-for-age (WAZ), body mass index (BMI) z-score (BAZ) and weight-for-height/length (WFH) z-score (WFHZ) were obtained using World Health Organization growth standards. The following definitions were used: (a) HAZ < −2 SD as stunted and −2 to −1 SD as at risk of stunting; (b) WFHZ < −3 SD as severe, −3 to < −2 SD as moderate wasting, and −2 to < +1 SD as normal; (c) WAZ −2 to −1 as at risk of underweight; (d) BAZ +1 to < +2 SD as at risk of and > +2 SD as overweight.

**Results:**

Of the 15,331 children surveyed, prevalence of stunting and at risk of stunting were 16.1 and 20.0%, severe and moderate wasting were 4.0 and 6.1%, while 21.1% was at risk of underweight. Prevalence of at risk of and overweight 14.2 and 7.3%, respectively. One in fifth (25.0%) children had at least one form of undernutrition (stunting and/or underweight/wasting). Of the 1,412 (13.2%) children reported to have risk factors of undernutrition, 47.2% had feeding difficulties, 44.8% had poor dietary intake and 8.0% had both. Boys, paternal height < 156 cm and poor dietary intake were significantly associated with stunting and/or wasting. Compared with children with no risk factors, children with feeding difficulties were more likely to be wasted (AOR: 1.48, 95% CI: 1.18–1.85), and had at least one form of undernutrition (AOR: 1.45, 95% CI: 1.25–1.69).

**Conclusions:**

In Malaysian children aged 1 to 5 years, dual burden of under- and overnutrition are common. Poor dietary intake and feeding difficulties were risk factors for undernutrition.

## Introduction

In 2020, it was estimated that 150 million and 45 million children under 5 years of age the world over were stunted and wasted, respectively, while 40 million or 5.7% of children of the same age group were overweight (1). The dual burden of undernutrition and overweight/obesity in children is also seen in Malaysia (2, 3). The proportion of children under 5 years in Malaysia who were underweight and stunted increased from 11.6 and 16.6%, respectively in 2011 to 14.1 and 21.8%, respectively in 2019 while the proportion of children who were overweight/obesity was 6.4% in 2016 and 5.6 % in 2019, respectively (2, 3).

The occurrence of dual burden of over- and undernutrition simultaneously has become a “new normal” (4, 5). Both over- and undernutrition can co-exist in the same individual, the same household, and the same population (5). At the individual level, for instance, an overweight child may be stunted or have micronutrient deficiency. In addition, over- and undernutrition can manifest in different stages over the lifetime of a person. A child may be undernourished in early childhood, followed by a rapid weight gain in late childhood and adolescence, and subsequently became overweight in adulthood with increased risk of non-communicable diseases (6, 7). This can even be passed across generations (8).

In addition, growth faltering in early life has been associated with increased risk of infection, impaired cognitive development and reduced educational performance in childhood and adolescence (9). This may eventually result in decreased economic productivity and an increased risk of non-communicable diseases in adulthood (10–14). Studies from low- and medium-income countries showed that a significant proportion of infants experienced growth faltering during the first 2 years of life (15, 16). Data from 51 national surveys from low- and middle-income countries also revealed that although linear growth deficit persisted between 18 months and 5 years, 70% of the absolute height deficits observed at 5 years of age occurred before age 2 years (17).

One of the most important cause of growth faltering in the first few years of life is food insecurity leading to inadequate energy and nutrient intake (18). Physical growth is also frequently restricted in children with malabsorption, food allergy or feeding problems (19–21). Knowledge on nutritional status in children and affecting factors is useful for both policymakers and healthcare providers of children in planning appropriate preventive and interventional measures. The aim of the present study was to determine the prevalence of stunting and underweight among children aged 1 to 5 years as well as factors associated with undernutrition in Malaysian children aged 1–5 years.

## Materials and methods

### Study design, setting, and sampling

Data were extracted from a childhood screening and counseling campaign, a cross-sectional nationwide survey conducted over a 4-month period (April-August) in 2019 covering 11 states and one territory in Malaysia. The current study was approved by the ethical committee of University Malaya Medical Center, Kuala Lumpur, Malaysia (MREC 202069-8734).

The sampling method employed was convenient, universal sampling. Parents of all children seeking healthcare service (i.e., vaccination or minor ailments) from the participating healthcare facilities during the campaign period who also fulfilled the study criteria were invited to take in the study. This was to avoid any potential misunderstanding among other parents that their children received different standard of care.

### Inclusive criteria and interview

Healthy children aged 1 to 5 years with no significant congenital abnormalities or health issues, or major intercurrent illnesses were eligible to take part. Verbal informed consent from accompanying parent(s) was obtained before the interview. Fathers, mothers, or both provided the consent of their children.

### Data collection

Information on gender, age, parental height, and factors affecting nutritional status (feeding issues, and dietary intake) were obtained *via* interviewer-administered questionnaire. Anthropometric measurements (weight and height) of children were taken by healthcare practitioners using standard procedures. For children aged 2 to 5 years, the height was measured to the nearest 0.1 cm using a stadiometer, and weight was measured to the nearest 0.1 kg using a digital weighing scale. Recumbent length was measured for children aged < 2 years using a length board, to the nearest 0.1 cm. Each measurement was performed twice, and the average was used in the analysis. Only children with complete anthropometric data were included in the analysis.

Anthropometric data for parents accompanying their child were obtained using methods described above for older children. Similarly, only parents with complete anthropometric data were included in the analysis.

### Feeding behavior and dietary intake

Questionnaire on feeding issues and food intake were modified from Kerzner et al. (22). Parents were asked the following questions: (a) if their child had feeding issues (easily distracted during mealtime by electronic gadgets, television, etc., being picky with food, limited variety of food, taking longer to finish their meal); and (b) if they were concerned about poor dietary intake (consuming limited choice and/or amount of food) in their child. Presence of one or more feeding issues listed in (a) was considered having feeding issues (22).

### Definition

Age and gender-specific z-score for length/height-for-age z-score (HAZ), weight-for-age z-score (WAZ), body mass index (BMI) z-score (BAZ) and weight-for-height/length (WFH) z-score (WFHZ) were used to assess the nutritional status of children aged 1 to 5 years and were analyzed using World Health Organization (WHO) Anthro software (version 3.2.2, 2011) (23). Children were classified according to HAZ as being stunted [< −2 standard deviations (SD)], at risk of stunting (−2 to −1 SD), and tall (≥ +2 SD), respectively (24). BAZ was used to classify overweight/obesity; at risk of overweight as BAZ +1 to < +2 SD and overweight as BAZ > +2 SD. Presence of and degree of wasting was classified using WFHZ; severely wasted (< −3 SD), moderately wasted (−3 to <-2 SD), and normal (−2 to < +1 SD). Finally, at risk of underweight was defined using WAZ as < −2 to −1.

Parental height was classified as short stature if the father's height was < 156 cm and the mother's height was < 145 cm, respectively (25).

For the ease of understanding, we stratify different combination of severity and forms of nutritional status into three categories: (a) “at least one form of undernutrition” includes children who were both underweight and stunted, underweight with any degree of height status, or stunted with any degree of weight status; (b) “at risk of one form of undernutrition” includes children who had WAZ of between −2 and −1 SD and not stunted (HAZ > −2 SD) and or HAZ of between −2 and −1 SD and not underweight (WAZ > −2 SD); and (c) “not at risk of any form of undernutrition” includes children who have both WAZ and HAZ > −1.

### Data analysis

Data were analyzed using the IBM SPSS Statistics 20.0 (IBM Corporation, New York, USA) and were cleaned and checked prior to statistical analysis. All variables [demographic features, nutritional status, parental height, and presence of risk factor(s)] were presented as mean ± SD for continuous variables, and frequency and percentage for categorical variables. Analysis were divided into two parts; (a) children with complete data on age, gender and anthropometric measurements were included in the analysis of growth status, (b) respondents with complete data on gender, age, anthropometric data, parental height, and factors affecting nutritional status were included in the analysis of risk factors for growth status.

Preliminary analyses including independent *t*-test for continuous variables and chi-square test for categorical variables were performed to compare the nutritional status of children aged 1 to 5 years by parental height and presence of risk factor. Pearson χ^2^ was used to determine the distribution of stunting and underweight by age groups while linear-by-linear association in χ^2^ was used to determine the trend of stunting/underweight across age group. Bonferroni test was used for *post hoc* analysis on the differences in parental height by child's height status, a significance level was set at 0.05.

Binary logistic regression analysis was employed to determine risk factors for stunting, underweight and presence of at least one form of undernutrition. Presence or absence of stunting or/and underweight were expressed as a dichotomous variable, category 0 as “not stunted (include at risk of stunting, normal and tall),” “not underweight (include at risk of underweight, normal, and overweight)” and category one as “stunted (<-2 SD)”, “underweight (<-2 SD)”. Variables on univariate analysis with a *p* < 0.25 or which were considered conceptually relevant were entered concurrently into the binary logistic regression model to assess its adjusted effect on the outcome, with years of data collection as covariates. Results were presented as adjusted odds ratios (AOR) with a 95% confidence interval (CI). Statistical significance was set at *p* < 0.05.

## Results

**Figure 1:**
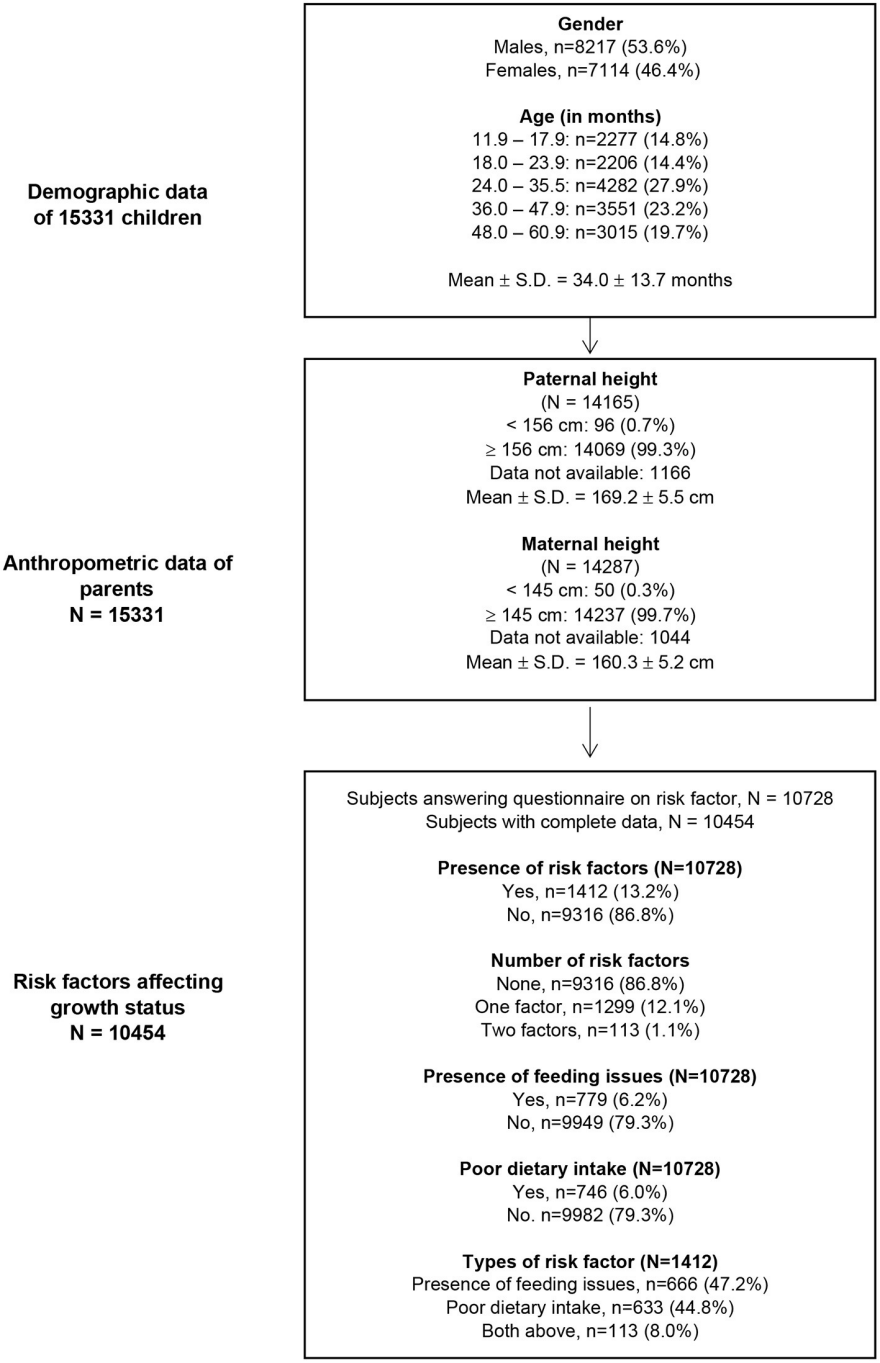
Demography, anthropometric data, and risk factors affecting growth status in Malaysian children aged 1 to 5 years.

Complete data on gender, age and anthropometric measurements were available in 15,331 (54% boys) children aged 1 to 5 years (Figure 1; Tables 1, 2), while 70% (*n* = 10,728) of the parents completed the questionnaire on feedings issues and dietary intake (Tables 3, 4). Parental height was available in 10,454 (68%) of the respondents (Figure 1). The mean (± SD) age of the 15,331 children at interview was 34.0 ± 13.7 months.

**Table 1:** Classification of growth status of 15,331 Malaysian children aged 1 to 5 years.

**Nutritional status**	**Male (*n* = 8,217)**	**Female (*n* = 7,114)**	**Total (*n* = 15,331)**
	***n* (%)**	***n* (%)**	***n* (%)**
**Length/height-for-age (Mean** **±SD)**	−0.57 ± 1.66	−0.39 ± 1.72	−0.49 ± 1.69
Stunting (< −2 SD)	1,417 (17.2)	1,044 (14.7)	2,461 (16.1)
At risk stunting (−2 to −1 SD)	1,669 (20.3)	1,395 (19.6)	3,064 (20.0)
Normal (>-1 to +2 SD)	4,651 (56.7)	4,147 (58.3)	8,798 (57.3)
Tall (> +2 SD)	480 (5.8)	528 (7.4)	1,008 (6.6)
**Weight-for-age (Mean** **±SD)**	−0.46 ± 1.27	−0.31 ± 1.19	−0.39 ± 1.24
Underweight (<-2 SD)	841(10.2)	531 (7.5)	1,372 (8.9)
At risk underweight (−2 to −1 SD)	1,851 (22.5)	1,376 (19.3)	3,227 (21.1)
Normal (> −1 to +2 SD)	5,306 (64.6)	5,003 (70.3)	10,309 (67.2)
Overweight (> +2 SD)	219 (2.7)	204 (2.9)	423 (2.8)
**BMI-for-age (Mean** **±SD)**	−0.16 ± 1.58	−0.10 ± 1.49	−0.13 ± 1.54
Severely thin (< −3 SD)	397 (4.8)	267 (3.8)	664 (4.3)
Moderately thin (−3 to < −2 SD)	532 (6.5)	395 (5.5)	927 (6.1)
Normal (−2 to < +1 SD)	5,476 (66.6)	4,966 (69.8)	10,442 (68.1)
At risk overweight (+1 to < +2 SD)	1,190 (14.5)	982 (13.8)	2,172 (14.2)
Overweight (≥+2 SD)	622 (7.6)	504 (7.1)	1,126 (7.3)
**Weight-for-length/height (Mean** **±SD)**	−0.23 ± 1.48	−0.15 ± 1.40	−0.1927 ± 1.45
Severe wasting (<-3 SD)	368 (4.5)	241 (3.4)	609 (4.0)
Moderate wasting (−3 to < −2 SD)	566 (6.9)	401 (5.6)	967 (6.3)
Normal (−2 to <1 +SD)	5,692 (69.3)	5,143 (72.3)	10,835 (70.7)
At risk overweight (+1 to +2 SD)	1,099 (13.4)	951 (13.4)	2,050 (13.4)
Overweight (> +2 SD)	492 (6.0)	378 (5.3)	870 (5.7)
**Classification of nutritional status**			
*At least one form of undernutrition* ^*A*^	2,299 (28.0)	1,539 (21.6)	(25.0)
Wasting (moderate and severe) and stunted	112 (1.4)	54 (0.8)	166 (1.1)
Wasting and at risk of stunting	190 (2.3)	88 (1.2)	278 (1.8)
Wasting only	503 (6.1)	371 (5.2)	874 (5.7)
Wasting and tall	129 (1.6)	129 (1.8)	258 (1.7)
Stunting only	892 (10.9)	582 (8.2)	1,474 (9.6)
Stunting and at risk of underweight	473 (5.8)	315 (4.4)	788 (5.1)
*At risk of one form of undernutrition* ^*A*^	2,186 (26.6)	1,787 (25.1)	3,973 (25.9)
At risk of stunting and at risk of underweight	569 (6.9)	468 (6.6)	1,037 (6.8)
At risk of underweight only	726 (8.8)	498 (7.0)	1,224 (8.0)
At risk of stunting only	871 (10.6)	791 (11.1)	1,662 (10.8)
At risk underweight and tall	18 (0.2)	25 (0.3)	43 (0.3)
At risk of stunting and overweight	5 (0.1)	5 (0.1)	10 (0.1)
*No risk of any form of undernutrition*	4,223 (51.4)	4,012 (56.4)	8,235 (53.7)

Length/height and weight of children were compared to the World Health Organization Child Growth Standards. A: Please refer to the text for definitions.

**Table 2:** Growth status of 15,331 children by age group and gender.

**Growth parameter**	**Age (months)**					**Pearson chi-square (*p*-value)**	**Linear-by-linear association (*p*-value)**
	**11.9–17.9**	**18.0–23.9**	**24.0–35.5**	**36.0–47.9**	**48.0–60.9**		
**Overall**							
**Length/height-for-age**							
Stunted (< −2 SD)	395 (17.3)	330 (15.0)	745 (17.4)	535 (15.1)	456 (15.1)	15.04 (0.005)	4.45 (0.035)
Non-stunted (≥−2 SD)	1,882 (82.7)	1,876 (85.0)	3,537 (82.6)	3,016 (84.9)	2,559 (84.9)		
**Weight-for-length/height**							
Wasted (< −2 SD)	307 (13.5)	199 (9.0)	401 (9.4)	354 (10.0)	315 (10.4)	33.47 (<0.001)	5.81 (0.016)
Non-wasted (≥−2 SD)	1,970 (86.5)	2,007 (91.0)	3,881 (90.6)	3,197 (90.0)	2,700 (89.6)		
**BMI-for-age**							
At risk of overweight (< −2 SD)	2,068 (90.8)	2,022 (91.7)	3,925 (91.7)	3,353 (94.4)	2,837 (94.1)	46.20 (<0.001)	36.24 (<0.001)
Overweight (≥−2 SD)	209 (9.2)	184 (8.3)	357 (8.3)	198 (5.6)	178 (5.9)		
**Male**							
**Length/height-for-age**							
Stunted (< −2 SD)	234 (19.3)	185 (15.6)	429 (18.1)	303 (16.3)	266 (16.6)	8.82 (0.066)	2.25 (0.133)
Non-stunted (≥−2 SD)	977 (80.7)	1,002 (84.4)	1,935 (81.9)	1,553 (83.7)	1,333 (83.4)		
**Weight-for-length/height**							
Wasting (< −2 SD)	173 (14.3)	112 (9.4)	254 (10.7)	212 (11.4)	183 (11.4)	15.56 (0.004)	1.39 (0.239)
Non-wasting (≥−2 SD)	1,038 (85.7)	1,075 (90.6)	2,110 (89.3)	1,644 (88.6)	1,416 (88.6)		
**BMI-for-age**							
At risk of overweight (< −2 SD)	1,092 (90.2)	1,085 (91.4)	2,181 (92.3)	1,752 (94.4)	1,485 (92.9)	21.29 (<0.001)	14.04 (<0.001)
Overweight (≥−2 SD)	119 (9.8)	102 (8.6)	183 (7.7)	104 (5.6)	114 (7.1)		
**Female**							
**Length/height-for-age**							
Stunted (< −2 SD)	161 (15.1)	145 (14.2)	316 (16.5)	232 (13.7)	190 (13.4)	8.39 (0.078)	2.09 (0.148)
Non-stunted (≥−2 SD)	905 (84.9)	874 (85.8)	1,602 (83.5)	1,463 (86.3)	1,226 (86.6)		
**Weight-for-length/height**							
Wasting (< −2 SD)	134 (12.6)	87 (8.5)	147 (7.7)	142 (8.4)	132 (9.3)	21.96 (<0.001)	21.96 (<0.001)
Non-wasting (≥−2 SD)	932 (87.4)	932 (91.5)	1,771 (92.3)	1,553 (91.6)	1,284 (90.7)		
**BMI-for-age**							
At risk of overweight (< −2 SD)	976 (91.6)	937 (92.0)	1,744 (90.9)	1,601 (94.5)	1,352 (95.5)	36.18 (<0.001)	23.20 (<0.001)
Overweight (≥−2 SD)	90 (8.4)	82 (8.0)	174 (9.1)	94 (5.5)	64 (4.5)		

BMI, body mass index; SD, standard deviation.

**Table 3:** Comparison between children who were stunted and non-stunted by parental height (*N* = 10,454).

**Parental height**	**Non-stunted**	**Stunted**	**Chi-square test**
	**(*n* = 8,965) (%)**	**(*n* = 1,489) (%)**	
**Paternal height**			
<156 cm	27 (0.3)	12 (0.8)	8.75
≥ 156 cm	8,938 (99.7)	1,477 (99.2)	(*p* < 0.01)
**Maternal height**			
<145 cm	22 (0.2)	6 (0.4)	1.19
≥ 145 cm	8,943 (99.8)	1,483 (99.6)	

**Table 4:** Association between growth status and the presence of risk factors for feeding or nutritional intake (*N* = 10,454).

**Risk factors**	**Length/height-for-age**			**Chi-square test**	**Weight-for-height**			**Chi-square test**
	**Stunting (*n* = 1,489)**	**At risk stunting (*n* = 2,053)**	**Not stunting (*n* = 6,912)**		**Severe wasting (*n* = 369)**	**Moderate wasting (*n* = 570)**	**No wasting (*n* = 9,517)**	
**Presence of risk factors**				10.95^**^				163.31^***^
No	1,272 (85.4)	1,746 (85.0)	6,049 (87.5)		294 (80.1)	438 (76.8)	8,335 (87.6)	
Yes	217 (14.6)	307 (15.0)	863 (12.5)		73 (19.9)	132 (23.2)	1,182 (12.4)	

** p < 0.01;

*** p < 0.001.

### Demographic features and growth parameters

Approximately six in 10 (57%) of the children surveyed had normal length/height-for-age while two thirds (67%) had normal weight-for-age. The proportion of children who were stunted and at risk of stunting were 16 and 20.0%, respectively while proportion of children who were underweight and at risk of underweight were 8.9 and 21%, respectively (Table 1). As compared to girls, boys were more likely to be stunted (17% vs. 15%, *p* < 0.001) and underweight (10 vs. 7.5%; *p* < 0.001). Using WFH to classify degree of wasting, 10.3% of the children were either severely (4.0%) or moderately wasted (6.3%, Table 1).

Approximately 70% of the children had normal BAZ, 14.2% were at risk overweight (BAZ 1 to < +2 SD) and 7.3% were overweight (BAZ ≥ 2 +SD; Table 1). In total, 21.5% were either at risk of overweight or overweight.

The percentage of children who had at least one form of undernutrition was 20.3% (Table 1). Of these, 4.6% were both underweight and stunted. Another 25.9% had at risk of one form of undernutrition. Only 53.7% did not have any form of undernutrition.

### Age and growth status

Table 2 shows the distribution of height, BMI and WFH by age group. There were significant differences in the proportion of children who were stunted (*p* = 0.005) and wasting (*p* < 0.001) across age groups. There was a reduction in the proportion of children who were stunted across age groups, children in the age group of 11.9–17.9 months (17.3%) and 24.0–35.5 months (17.4%) were more likely to be stunted than the other age groups (linear-by-linear association χ^2^ = 4.45, *p* = 0.035; Table 2).

Children in the age group of 11.9–17.9 months (13.5%) were significantly more likely to have wasting than children in other age groups (linear-by-linear association χ^2^ = 5.81, *p* < 0.001; Table 2). No gender difference was observed in the proportion of children who were stunted across different age groups. For wasting, the proportion of boys (*p* < 0.001) and girls (*p* < 0.001) who were wasted at 11.9–17.9 months was significantly higher compared to children in other age groups (Table 2).

There were significant differences in proportion of children who were at risk of overweight and being overweight (χ^2^ = 46.20; *p* < 0.001) (Table 2). Children in the age group of 11.9–17.9 months were more likely to be overweight than the other age groups. Similar significant differences in the proportion of children who were overweight were also observed according to gender across different age groups.

There is a significant reduction in the proportion of children who were overweight across age groups (Table 2). Children in the younger age groups were more likely to be overweight as compared to children in the older age group. Similar trend was observed in the proportion of children being overweight for both genders across age groups (Table 2).

### Parental height and growth status

The mean (± SD) paternal height was 169.22 ± 5.54 cm with a great majority (99%) had a height ≥ 156 cm (Figure 1). The mean maternal (± SD) height was 160.27 ± 5.18 cm with a majority (99%) had a height ≥ 145 cm. There was a small but significant difference in the mean paternal height between stunted and non-stunted children.

Table 3 shows the relationship between presence of stunting and paternal and maternal height status. As compared to children who were not stunted, children who were stunted were significantly more likely to have a father who were short (*p* < 0.01; Table 3). However, no significant association were observed between presence of maternal short stature and stunting in children.

### Feeding behavior, dietary intake, and growth status

Of the 10,728 (70% of 15,331) parents who responded to the questionnaire on feeding issues, 13.2% (*n* = 1412) reported presence of concerns on feeding and/or dietary intake of their child (Figure 1). Feeding issues and poor dietary intake were equally common, being reported in 7.3% (*n* = 779) and 7.0% (*n* = 746) children, respectively. Only 1.1% (*n* = 113) of the children were reported to have both feeding issues and poor dietary intake.

Children who were stunted (14.6%) or at risk of stunting (15.0%) were more likely to have feeding issues and/or poor dietary intake than children who were not stunted (12.5%; *p* < 0.01; Table 4). Similarly, children who were severely wasted (19.9%) or moderately wasted (23.2%) were more likely to have feeding issues and/or poor dietary intake than children who were not wasted (12.4%; *p* < 0.001; Table 4).

Factors associated with stunting, wasting as well as presence of at least one form of undernutrition are shown in Table 5, respectively. Male gender, paternal height ≤ 156 cm, as well as presence of feeding issues and poor dietary intake were all significantly associated with stunting and wasting (Table 5). Similarly, male gender, paternal height ≤ 156 cm, and poor dietary intake were significantly associated with children who have at least one form of undernutrition (Table 5).

**Table 5:** Factors affecting nutritional status of 10,454 Malaysian children aged 1–5 years.

	**Stunting^†^ (*n* = 1,489)**	***p*-value**	**Wasting^‡^(*n* = 815)**	***p*-value**	**At least one form of undernutrition (*n* = 3,848)^Ψ^**	***p-*value**
	**AOR [95% CI]**		**AOR [95% CI]**		**AOR [95% CI]**	
**Gender**						
Male	1.12 [1.01–1.26]	0.04	1.52 [1.32–1.75]	<0.001	1.24 [1.14–1.34]	<0.001
Female	1.00		1.00		1.00	
**Paternal height (cm)**						
<156	2.76 [1.38–5.55]	0.01	2.34 [1.01–5.43]	<0.05	2.35 [1.23–4.52]	<0.01
≥ 156	1.00		1.00		1.00	
**Maternal height (cm)**						
<145	1.30 [0.51–3.32]	0.58	0.95 [0.28–3.27]	0.93	1.50 [0.70–3.22]	0.30
≥ 145	1.00		1.00		1.00	
**Presence of feeding issues**						
Yes	0.79 [0.63–0.99]	0.04	1.48 [1.18–1.85]	0.001	1.45 [1.25–1.69]	<0.001
No	1.00		1.00		1.00	
**Poor dietary intake**						
Yes	1.41 [1.15–1.71]	0.001	2.68 [2.18–3.28]	<0.001	1.95 [1.67–2.27]	<0.001
No	1.00				1.00	

Reference group for dependent variables:

† Not stunted children (n = 8,965);

‡ Not underweight children (n = 9,639).

Reference group for dependent variables:

Ψ Normal children (n = 6,606);

^‡^Undernourished children: children with at least one form of undernutrition (n = 3,848).

AOR, adjusted odds ratio; CI, confidence interval.

## Discussion

In the current study on 15,331 Malaysian children younger than 5 years of age, the rate of stunting and wasting was 16.1 and 10.4%, respectively while 1.1% were both stunted and wasted. Almost one in three young Malaysian children were found to be either stunted or at risk of stunting while three in 10 children were either underweight or at risk of underweight. The present study confirms the findings of the Malaysian National Health and Morbidity Survey (NHMS) in 2019 where the stunting rate of 21.8% and an underweight rate of 14.1% were noted in children under 5 years (3). Both studies confirmed the significant magnitude of undernutrition in young Malaysian children.

In addition to the data by NHMS the current study showed 20% of young Malaysian children were at risk of stunting and 21% were at risk of underweight. It is important to detect both underweight and stunting at an earlier stage before growth status deteriorated further. It has been shown that when appropriate measures were taken in children who were at risk of undernutrition, the growth parameters can be reversed (26).

Our observation that undernutrition in children under five is more likely to affect boys than girls is consistent with the findings of a meta-analysis on gender differences in undernutrition which also showed boys had higher odds of stunting and being wasted than girls (27). At present, there is no satisfactory explanation for this observed gender difference but there may be implications for nutrition policy and practice (27).

Another systematic analysis showed that although male disadvantage in linear growth is most evident in the first years, the gender gap has largely disappeared by the age of 4 years and in some countries, the gap has been reversed (28). The result of our study is somewhat different. We observed a reduction in the proportion of children who were stunted in both gender across age groups.

We also found that poor dietary intake was a risk factor for underweight and/or stunting in children. This is not surprising as poor dietary intake may impede the process of catch-up growth, which in turn leads to underweight, stunting or both (1). Similarly, a study in urban Malaysian children found that stunting was associated with a diet with higher energy density, whereby a higher dietary energy density was associated with lower carbohydrate, sugar, vitamins C and D, and calcium intakes but higher in fat, fiber, iron, and folate intakes (29). Thus, in addition to increasing energy intake, it is also important to attain minimal meal frequency and minimum dietary diversity. High energy intake alone does not necessarily guarantee good bone growth and good gain in height. In setting where food intake is adequate, optimum dietary diversity which include optimum substrate for bone growth, including adequate vitamin D, calcium, and phosphate intake is important.

The present study also noted that children who had feeding issues were more likely to being underweight and stunted, albeit less significant for stunting. Persistent feeding difficulties may result in an imbalanced diet or insufficient nutrients intake, leading to weight loss (30). However, we did not analyse whether having just one or both factors affected the child's height status as number of children reported to have both factors were small. A study of healthy children (12–36 months) by Lee et al. showed that children who had food refusal were more likely to have growth faltering, while children with limited variety of food intake were less likely to have short stature (31).

Surprisingly, we found that although short paternal stature was a significant risk factor for undernutrition in children aged 1 to 5 years, no significant association was noted between maternal height and child undernutrition. In this study, children were 2.35–2.76 times more likely to being stunted and/or underweight if having a father who was short. This could be due to the cumulative effect of the interaction between genetic and early-life environmental factors (i.e., nutrition and disease occurrence), which first affect fathers, and then further affect the growth of their children (32). This association may indicate an intergenerational transmission of stunting from fathers to children. However, our results are inconsistent with the findings of previous cross-sectional and longitudinal studies (16, 32–34). A recent meta-analysis of Demographic and Health Surveys (2007–2018) in 35 low- and middle-income countries showed that parental stature was protective against child undernutrition, and maternal short stature had a stronger association with child undernutrition as compared to paternal short stature (32). Our finding of a lack of association between maternal short stature and the child's height was surprising. Recently, however, other maternal factors including interpregnancy interval and maternal anemia have also been found to be important determinants of child's nutritional status and height (35). Further studies which include maternal parity and interpregnancy internal in addition to maternal height status are needed to clarify the effect on child's height status may help to explain the finding of our study.

The present study also confirms the Malaysian NHMS 2019 study where 21.5% of children were either at risk of overweight or overweight (2, 3). This finding reaffirms similar findings in many countries where dual burden of under- and overnutrition in children are common (4, 5).

The strength of this study is it was a nationwide survey with a large sample size. The anthropometric measurements of the children were collected by trained personnel. Secondly, the findings of this study ascertain the current gaps in knowledge on childhood nutritional problems and the risk factors of poor dietary intake and feeding issues. However, a cause-and-effect relationship between the presence of risk factors and childhood undernutrition cannot be established as it was a cross-sectional study.

There are several shortcomings in the present study. Firstly, this study did not fully account for socioeconomic factors, such as household income and parental education. Future studies are needed to improve the understanding on the underlying mechanisms of childhood undernutrition and the potential context-specific interactions. In addition, the data on risk factors of undernutrition were based on parental self-report which may have potential response biases. The definition of feeding difficulty and inadequate intake were not precise enough. This was decided after considering the diverse nature of the interviewees with different educational level and cultural background. Future study should attempt to address this limitation.

In conclusion, undernutrition among children aged ≤ 5 years is of major concern in Malaysia. About one in five children in this study reported having at least one form of undernutrition (stunting and/or underweight). Preventive measures such as promotion of breastfeeding and appropriate feeding practices in early childhood are essential in shaping healthy dietary intake to attain and maintain optimal growth. Nutritional intervention strategies emphasizing healthy diets and appropriate feeding practices would help to promote optimal early childhood growth. In addition, regular monitoring of the child's growth status, diets and feeding is recommended as part of the child's routine care and anticipatory guidance.

## Data availability statement

The raw data supporting the conclusions of this article will be made available by the authors, without undue reservation.

## Ethics statement

The studies involving human participants were reviewed and approved by Medical Research Committee, University Malaya Medical Center, Kuala Lumpur, Malaysia. Written informed consent to participate in this study was provided by the participants' legal guardian/next of kin.

## Author contributions

WL, MJ, KK, JK, TN, AS, YF, AZ, and HC conceived the research idea and designed the data collection. WL and HC supervised the cleaning and analysis of the data. WL interpreted the data and wrote the first draft of the manuscript. MJ and HC gave critical input to the manuscript. All authors read and agreed to the final version of the manuscript.

## Funding

The present work was made possible by an unrestricted educational grant from Abbott Nutrition Malaysia. None of the staffs from Abbott Nutrition Malaysia were involved in inception, planning, data collection and analysis, and manuscript preparation. None of the authors received any honorarium for the purpose of the project from the sponsor.

## Conflict of interest

The authors declare that the research was conducted in the absence of any commercial or financial relationships that could be construed as a potential conflict of interest.

## Publisher's note

All claims expressed in this article are solely those of the authors and do not necessarily represent those of their affiliated organizations, or those of the publisher, the editors and the reviewers. Any product that may be evaluated in this article, or claim that may be made by its manufacturer, is not guaranteed or endorsed by the publisher.
